# Modification of Polypropylene Fibers with Sodium Silicate: Enhancement of Pozzolanic Properties in Cement-Based Systems

**DOI:** 10.3390/polym17233206

**Published:** 2025-12-01

**Authors:** Yahya Kaya, Petek Balcı, Süleyman Özen, Ali Mardani, Ali Kara

**Affiliations:** 1 Department of Civil Engineering, Faculty of Engineering, Bursa Uludag University, 16059 Bursa, Turkey; yahyakaya00@gmail.com; 2 Department of Polymer Materials, Faculty of Arts and Sciences, Bursa Uludağ University, 16059 Bursa, Turkey; petekbalci@outlook.com; 3 Department of Civil Engineering, Faculty of Engineering and Natural Sciences, Bursa Technical University, 16310 Bursa, Turkey; suleyman.ozen@btu.edu.tr; 4 Department of Physical Chemistry, Faculty of Arts and Sciences, Bursa Uludag University, 16059 Bursa, Turkey; akara@uludag.edu.tr

**Keywords:** PP fiber, cementitious system, sodium silicate, pozzolanic properties

## Abstract

This study investigates the effect of sodium-silicate-based chemical surface modification of polypropylene (PP) fibers on the mechanical and fresh-state properties of cementitious composites. The proposed method introduces silanol and siloxane groups onto the PP surface through a radical-assisted chlorination route, aiming to enhance fiber–matrix interfacial bonding. Modified fibers increased the polycarboxylate ether (PCE) demand by 100% compared to the control mixture, while unmodified PP fibers caused a 58% increase at equivalent workability. The incorporation of PP fibers resulted in limited changes in compressive strength (1-7%), whereas silicate-modified fibers led to notable late-age flexural strength gains of 10% (28 days) and 17% (56 days). Scanning Electron Microscopy-Energy Dispersive X-ray Spectroscopy (SEM-EDX) and Fourier Transform Infrared Spectroscopy (FTIR) analyses confirmed successful surface functionalization, while the heterogeneous silicate deposition still contributed positively to interfacial transition zone (ITZ) performance. Overall, sodium-silicate-modified PP fibers improve flexural behavior and interfacial bonding in cement-based systems, offering a promising approach for enhanced mechanical performance and sustainability.

## 1. Introduction

Sustainability and durability have become increasingly critical considerations in the construction industry, particularly given the substantial carbon footprint associated with conventional cement production and the accelerating demand for long-lasting infrastructure [1,2]. Cementitious materials, though widely used, inherently suffer from limitations such as low fracture toughness, susceptibility to cracking, high permeability, and progressive deterioration under environmental and mechanical stressors [3]. These shortcomings can compromise structural integrity and lead to increased maintenance requirements over a structure’s service life [4,5,6]. Therefore, developing reinforced cementitious systems with enhanced durability and reduced environmental impact has become a central research focus, motivating the use of supplementary cementitious materials, polymeric fibers, and chemical modifications to improve long-term performance.

Among reinforcement strategies, polypropylene (PP) fibers have attracted significant attention due to their low cost, low density, chemical stability, and effectiveness in controlling plastic shrinkage cracking. However, the inherently hydrophobic and chemically inert surface of PP fibers severely limits their interfacial bonding with the cement matrix, resulting in suboptimal stress transfer and restricted improvements in mechanical properties [7,8]. For this reason, researchers have increasingly explored surface modification approaches to improve the compatibility and bonding performance of PP fibers in cement-based systems. Chemical modification techniques—including oxidation, chlorination, and silane coupling have been shown to increase the surface energy, hydrophilicity, and reactivity of fibers. For example, Hu and Ma [9] reported that silane-functionalized PP fibers exhibited a reduced water contact angle and enhanced sulfate resistance due to improved fiber-matrix adhesion. Similarly, Signorini et al. [10] demonstrated that silica nano-coatings significantly increased the surface roughness and bonding capacity of PP fibers without altering their bulk morphology, making the method suitable for industrial-scale application.

Parallel findings from polymer composite research also underscore the value of silicon-based chemistry in overcoming the low-energy properties of the PP surface. Han et al. [11] demonstrated that a combination of plasma activation followed by silane treatment markedly increased interfacial strength between fibers and a PP matrix, reinforcing the importance of chemically introducing reactive functional groups to the polymer surface. Physical and photo-induced modification techniques, such as UV-LED, laser ablation, and corona discharge, have also been employed to enhance the hydrophilicity and surface activation of PP fibers while maintaining their structural integrity [12]. These studies collectively demonstrate that the effectiveness of PP fibers in cementitious systems is primarily determined by their surface chemistry, wettability, and interfacial transition zone (ITZ) interactions.

Complementing fiber modification strategies, sodium silicate has emerged as an environmentally favorable pozzolanic additive due to its ability to react with calcium hydroxide to form secondary calcium silicate hydrate (C-S-H), thus improving matrix densification, reducing permeability, and enhancing mechanical strength [13]. Pozzolanic reactions also contribute to long-term durability by refining the pore structure and mitigating chemical ingress. Previous studies incorporating PP fibers with pozzolans—such as silica fume, volcanic ash, and polymineral additives have demonstrated synergistic effects in terms of improved mechanical performance, crack resistance, and durability [14,15]. However, the majority of these studies focus on modifying the cement matrix rather than directly engineering the fiber surface, leaving a critical gap in understanding the interfacial behavior of chemically functionalized PP fibers.

Despite advancements in fiber and matrix modifications, there remains a limited understanding of the direct chemical grafting of sodium silicate onto polypropylene fibers and the resulting implications for ITZ development and mechanical performance. Existing studies predominantly rely on physical treatments or silane-based mechanisms, whereas the potential of a multi-step chemical functionalization route capable of producing covalently bonded silanol and siloxane structures on PP has not been explored in cementitious applications.

The novelty of the present study lies in the development and application of a radical-assisted chlorination and sodium-silicate grafting process-used for the first time in the literature-to chemically functionalize PP fiber surfaces for enhanced performance in cementitious systems. This approach introduces reactive silicate-based groups directly onto the PP surface, promoting improved hydrophilicity, increased fiber-matrix chemical affinity, and localized ITZ densification. By isolating the effect of sodium-silicate-modified fibers under constant mix parameters, this study provides a mechanistic understanding of how chemically engineered fiber surfaces influence rheological behavior, superplasticizer demand, compressive and flexural strengths, and interfacial bonding in cementitious composites. These contributions address a critical knowledge gap and offer a promising direction for the design of high-performance, fiber-reinforced, and sustainability-oriented cement-based materials.

## 2. Materials and Methods

### 2.1. Materials

#### 2.1.1. Cementitious System Materials

CEM 42.5 R-type cement was used as a binder in this study. The chemical composition, physical, and mechanical properties of the cement provided by the manufacturer are given in Table 1.

**Figure 1:**
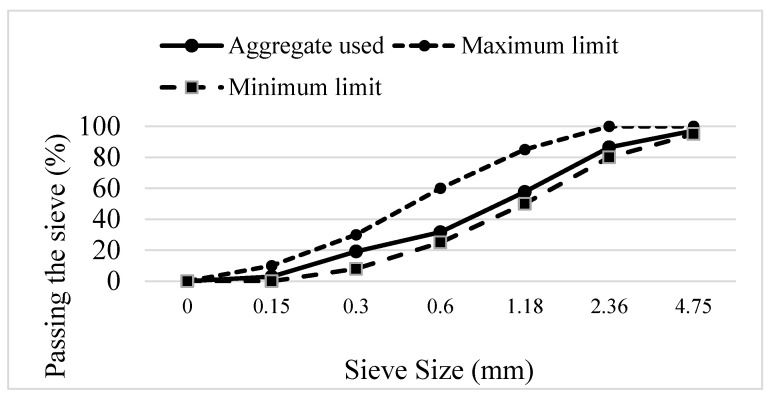
Grading Curve of Fine Aggregate Used and ASTM C33 Standard [16] Limits.

Crushed limestone sand (0–4 mm) was used, with a water absorption of 2.03% and specific gravity of 2.6. Its particle size distribution fell within ASTM: C33 [16] limits, as illustrated in Figure 1.

A single polycarboxylate ether (PCE) admixture was used, and its dosage is reported both as a percentage by weight of binder (bwob) and grams per cubic meter (g/dm^3^), enabling mixture-to-mixture comparability at a fixed workability. Some properties of the water-reducing admixture provided by the manufacturer are given in Table 2.

Within the scope of the study, 6 and 12-mm-long polypropylene fiber was used. Fiber volume fraction fixed at 0.5 vol%. The fiber volume replaced an equal volume of fine ag-gregate. Some properties of the fiber used, provided by the manufacturer, are given in Table 3.

#### 2.1.2. Chemical Synthesis

##### Materials for the Synthesis of Modification of PP Polymer Surface with Sodium Silicate

In the synthesis; 1M Sigma-Aldrich Potassium Persulfate (CAS Number: 7727-21-1), 0.1M Sigma-Aldrich Ethyl Chloroformate (CAS Number: 541-41-3), 95 mL Purified Water (NUVE ND-12 Water Purifier), 5 mL Chem-Pure Sodium Silicate (CAS Number: 1344-09-8) and 10 g polypropylene fibers, which have been shown to effectively enhance the post-cracking behavior and ductility of cementitious systems in recent investigations, were supplied by (Polisan Chem.) were used. All reactions were conducted under a fume hood with appropriate personal protective equipment (PPE). In addition, unmodified PP fibers (also referred to as the ‘blank’ or control group) were prepared.

### 2.2. Methods

#### 2.2.1. Synthesis Method

##### Synthesis of Modification of PP Polymer Surface with Sodium Silicate

This method, which is used for the first time in the literature, includes an oxidation process with radical initiator potassium persulfate and the addition of sodium silicate to the fiber surface. This process is designed to change the chemical structure of polypropylene fibers and to add new functional groups. In addition, the solubility and process conditions used in this method allow the surface properties of polypropylene to be modified.

**a.**  **Reactor Preparation and Addition of Reagents**

**Figure 2:**
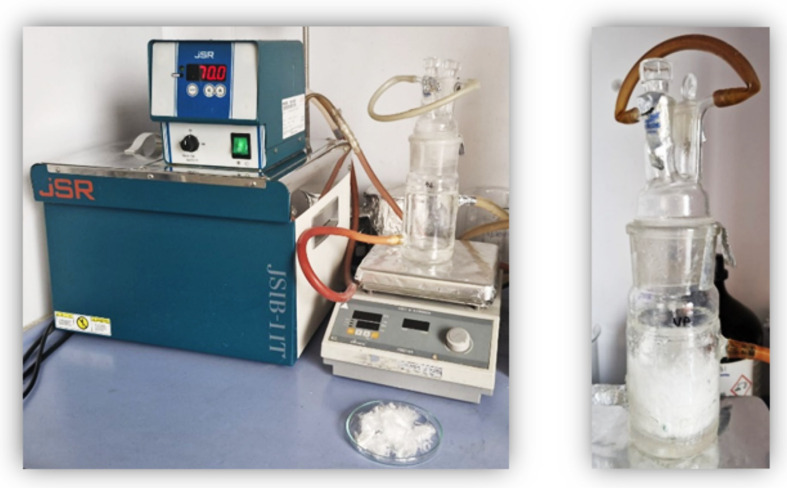
Pre-synthesis and synthesis image of modification of the PP polymer surface with Sodium Silicate.

**Steps:** First, potassium persulfate (KPS) radical initiator was prepared with 95 mL of pure water at a concentration of 1M. Potassium persulfate will cause oxidation reactions to occur on the surface of polypropylene fibers by creating free radicals. In addition, 0.1 M ethyl chloroformate was added to the reaction medium to prepare the chemical environment required for chlorination and surface functionalization of polypropylene. These chemicals were mixed to ensure homogeneous solubility in the reactor (Figure 2).

**b.**  **Surface Modification of Polypropylene Fibers**

In the first stage, 6 mm and 12 mm sized PP fibers were added to the reactor by weighing 10 g into separate reactors. This initial step aims to create active sites on the inert PP fiber surface through radical-induced oxidation, facilitating subsequent chemical bonding. This stage is critical for the modification of PP fibers. In order for the fibers to react homogeneously and change their surface, they were mixed at 1000 rpm and 70 °C for 45 min.

High-speed mixing facilitated the effect of radical initiators on the fiber surfaces, while temperature control optimized the reaction rate. During this process, ethyl chloroformate was converted into reactive chlorine groups on the surface of the polypropylene fibers.

**c.**  **Intermediate Step**

During the reaction, chlorination reactions begin on the surface of the polypropylene fibers, and temperature and time must be carefully adjusted for these reactions to proceed in a controlled manner. Additionally, free radicals formed by the action of potassium persulfate cause bond breaks and functional group additions to the polypropylene molecules, thereby increasing the surface’s chemical reactivity.

**d.**  **Modification with Sodium Silicate**

The next step of the surface modification is the addition of 5 mL of sodium silicate to the reactor. Sodium silicate was introduced to graft silanol (Si-OH) groups onto the activated fiber surface, thereby enhancing its hydrophilicity and potential for pozzolanic reactions with cement hydrates. This step was carried out to increase the surface energy and wettability of the fibers, introducing silanol/siloxane groups that enhance chemical affinity and ITZ bonding. The addition of sodium silicate enabled the silica groups on the surface to bind to the fibers, thereby enhancing the functional properties of the fibers.

**e.**  **Reaction Time and Mixing**

After this modification, the reaction process was continued for a total of 4 h. The reaction pH was controlled throughout the process (approximately 1–2 during ethyl chloroformate addition and raised to about 11 after sodium silicate dosing), while the temperature was maintained at 70 ± 2 °C with a mixing speed of roughly 1000 rpm, resulting in a total reaction time of 4 h. During this period, chemical bonds and functional groups were effectively established on the surface of polypropylene fibers, and chlorination and silicate modification occurred on the surface.

**f.**  **Drying and Subsequent Processes**

**Figure 3:**
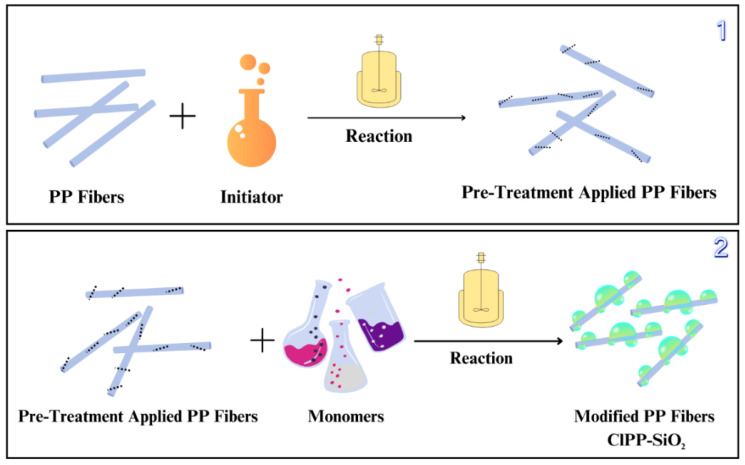
Synthesis steps: Steps of pre-treatment of PP fibers (**1**), application of the modification process on pretreated PP fibers (**2**).

After the modification process was completed, the obtained active polypropylene fibers were dried in an oven at 80 °C to prevent the formation of agglomeration and to ensure homogeneous drying. During the drying process, the fibers must be mixed. This mixing process was carried out every 20 min to ensure that the fibers dried without sticking to each other. It was observed that drying at low temperature maximized the effectiveness of the modification by preserving the properties of the fibers. The schematic view of the synthesis is given in Figure 3.

#### 2.2.2. Chemical Reactions

Polypropylene is chemically inert, so it cannot be modified directly by grafting silicate onto its non-polar surface. To solve this, a special functionalization step is used as a ‘chemical bridge.’ The process includes these stages:

1. *Radical Activation*: The reaction initiates when potassium persulfate (K_2_S_2_O_8_) thermally decomposes, making sulfate radicals (SO4^·−^). These radicals are strong hydrogen abstractors, and they attack the tertiary carbon atoms of the PP backbone to create macro-radicals (PP·) on the surface. This step weakens the otherwise inert polymer surface [14,17] (Equation (1)).
2. *Chlorination (The Bridge Step)*: Next, ethyl chloroformate (C_2_H_5_OCOCl) reacts with the newly created PP· radicals (Equation (2)). This step adds reactive chlorine groups to the fiber, forming the CIPP intermediate. The C-Cl bond acts as a reactive site susceptible to nucleophilic attack, unlike the stable C-H bonds of the original polymer. Ethyl chloroformate was specifically selected for this step to facilitate surface-selective functionalization within the aqueous radical polymerization system while preserving the structural integrity of the polypropylene fibers [18]. This makes the surface chemically active and ready for further reaction. Alternative chlorinating agents (e.g., SOCl_2_ or Cl_2_ gas) were excluded as they are incompatible with the aqueous reaction medium or induce excessive polymer degradation [19].
3. *Silicate Grafting*: In the final stage, sodium silicate (Na_2_SiO_3_) is added. The hydrolyzed silanol groups (Si-OH) undergo a nucleophilic substitution reaction with the chlorinated sites (CIPP). This results in the covalent attachment of a silicate layer (CIPP-SiO_2_) (Equation (4)). Consequently, the fiber surface transforms from hydrophobic to hydrophilic, gaining the necessary chemical affinity to bond with the cementitious matrix [12,13,14].

The governing chemical equations are:

(1) $$K_{2}S_{2}O_{8}\;\to \;2{\mathrm{SO}}_{4}\;\;\;(\mathrm{Sulfate}\;\mathrm{radicals})$$

(2) $$C_{2}H_{5}\mathrm{OCOCl}+\cdot \mathrm{PP}\;\to C_{2}H_{5}\mathrm{OCO}\cdot \mathrm{CIPP}\;(\mathrm{Chlorination}\;\mathrm{of}\;\mathrm{PP})$$

(3) $$\mathrm{PP}+C_{2}H_{5}\mathrm{OCOCl}\;\to \;\mathrm{CIPP}\;+\;R\;(\mathrm{Chlorine}\;\mathrm{and}\;\mathrm{functional}\;\mathrm{group}\;\mathrm{formation})$$

(4) $${\mathrm{Na}}_{2}{\mathrm{SiO}}_{3}+\mathrm{CIPP}\;\to \;\mathrm{CIPP-Si}O_{2}\;(\mathrm{Silica}\;\mathrm{functionalization}\;\mathrm{of}\;\mathrm{PP})$$

#### 2.2.3. Characterization Processes

After the synthesis process, the surface properties of the modified polypropylene fibers were characterized. All characterization analyses were performed using the equipment located at the Central Research Laboratory of Bursa Technical University (MERLAB, Bursa, Turkey). FT-IR (Fourier Transform Infrared Spectroscopy) and SEM-EDX (Scanning Electron Microscopy-Energy Dispersive X-Ray Spectroscopy) analyses were used for characterization. FT-IR analysis confirmed the changes in functional groups related to chlorination and silicate modification on the surface of polypropylene fibers. SEM-EDX provided information about the surface morphology and distribution of elemental components, thus the location and interactions of sodium silicate and chlorine on the surface were observed; the surface images of the fibers were obtained using a Zeiss/Gemini 300 microscope, while the chemical compositions of the coating materials on the surface were analyzed by EDS (Energy Dispersive Spectroscopy) method via a Bruker/XFlash 6100 device. FT-IR analysis was performed in the range of 4000–400 cm^−1^ with a resolution of 4 cm^−1^ using 32 scans, and the spectra were baseline-corrected and normalized to the ~2916 cm^−1^ CH_2_ band. SEM-EDX was conducted at 15 kV with a 10 mm working distance and approximately 60 s live time, and elemental data were reported as normalized wt% based on measurements from at least three regions per fiber type.

### 2.3. Preparation of Mixtures in Cementitious Systems

Mortar mix calculations were performed for a 1 dm^3^ volume. Mortar mixes were produced in accordance with ASTM C109 Standard [20]. The water/binder ratio and slump flow were kept constant at 0.485 and 200 ± 20 mm, respectively, for all mixes.

In addition to the fiber-free control mix, 4 more batches of mortar mixes were prepared using 6 and 12-mm-long PP and modified PP fibers. In the mixtures containing fiber, fiber was substituted with aggregate at 0.50% of the total volume. The authors determined the fiber volume used in the study in line with preliminary studies [17,21]. The naming of the mixtures was based on the type and length of the fibers. For example, the mixture containing 12 mm long unmodified polypropylene fibers was denoted as 12-PP, while the mixture containing 6 mm long silicate-modified PP fibers was named 6-M-PP. A water-reducing admixture was added to the mix at varying ratios to achieve the desired slump flow. Within the scope of the study, the production of 5 different mortar mixes was planned. The amount of material used in the production of 1 dm^3^ mortar mixes and the slump-flow values are shown in Table 4. The specimens were kept in lime-saturated water with a temperature of 20 °C until the test day.

#### Cemented System Tests

Slump-flow values, compressive strength, and flexural strength of all mortar mixtures were determined in accordance with ASTM C1437 [22] and EN 196-1 standards [23], respectively. Compressive strength tests were performed using a UTEST UTC-5600 automatic compression testing machine with a maximum load capacity of 3000 kN. The loading rate was controlled at 2.4 kN/s as specified in EN 196-1. Flexural strength tests were conducted using the same testing system, equipped with a three-point bending fixture. Prismatic specimens (40 × 40 × 160 mm) were tested under a constant loading rate of 50 ± 10 N/s, and the span-to-depth ratio was maintained at 1:3 (100 mm span length) according to EN 196-1 [23] requirements. For both flexural and compressive tests, three samples were tested for each mixture at each curing age (7, 28, and 56 days), and their average values are given.

## 3. Results and Discussion

### 3.1. Fiber Characterization

- FTIR Analyses

**Figure 4:**
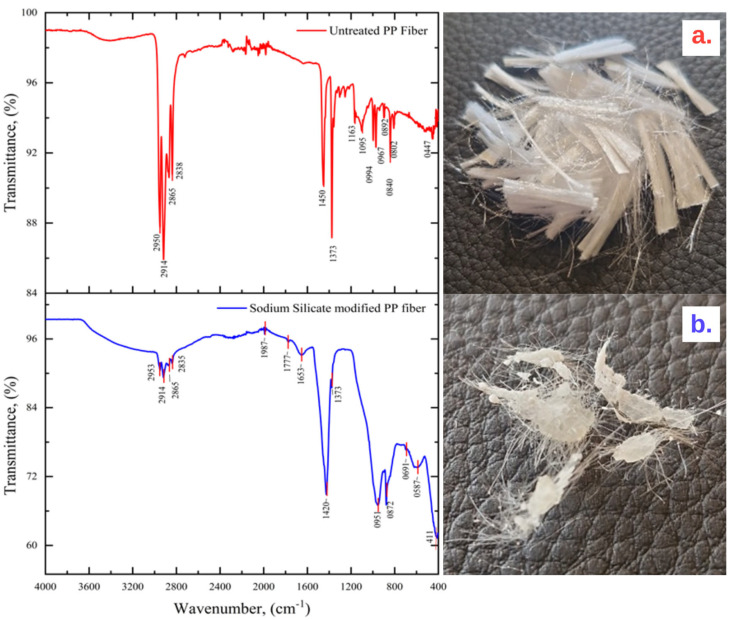
FTIR spectra of (**a**) untreated (unmodified) PP fiber, showing characteristic C-H absorption bands, and (**b**) sodium silicate treated PP fiber, showing new absorption bands for O-H (silanol groups, ~3400 cm^−1^) and Si-O-Si (~951 cm^−1^), confirming surface modification.

The FTIR spectra of untreated and sodium-silicate-treated polypropylene (PP) fibers are presented in Figure 4a,b. The spectrum of the unmodified PP fibers exhibited characteristic absorption bands typical of polypropylene: stretching vibrations of –CH_2_ and –CH_3_ groups at 2953–2835 cm^−1^, bending vibrations at 1420–1373 cm^−1^, and crystalline isotactic PP-related bands near 951–872 cm^−1^. These results are consistent with previous studies on the molecular structure of PP [24,25]. Following surface treatment with sodium silicate, new absorption features appeared in addition to the original PP signals. A broad band centered around 3400–3200 cm^−1^ was attributed to O–H stretching vibrations of silanol groups and physically adsorbed water. The strong band in the region of 951 cm^−1^ corresponded to Si–O–Si asymmetric stretching, while peaks detected at 872–411 cm^−1^ were assigned to Si–O bending vibrations. These features are indicative of silicate species deposited on the PP fiber surface [26,27]. The simultaneous presence of PP-specific vibrations and additional silicate-related bands demonstrates that the modification process altered the fiber surface without damaging the bulk polymer backbone. Similar findings were reported for silane- and silicate-modified PP fibers used in cementitious composites [28]. Consequently, the introduction of silanol and siloxane functionalities is expected to enhance the chemical affinity of PP fibers toward the cement hydration products, thereby promoting stronger interfacial bonding.

b. SEM-EDX Analysis

**Figure 5:**
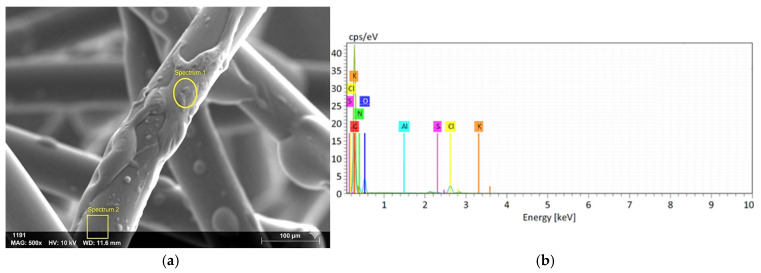
(**a**) SEM image and (**b**) EDX graph of PP fiber treated with Sodium Silicate.

**Figure 6:**
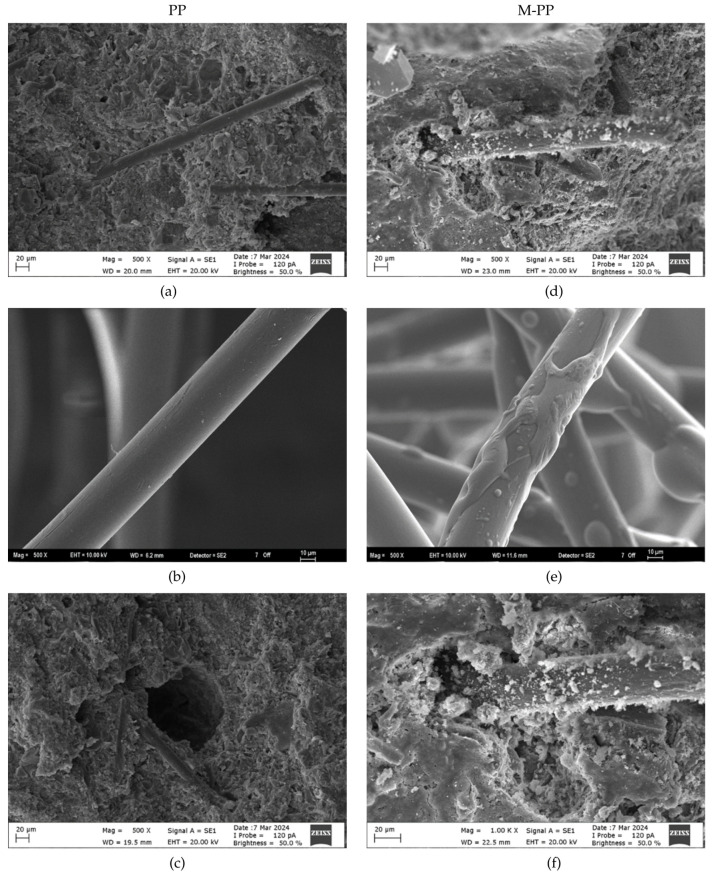
SEM images of (**a**–**c**) PP and (**d**–**f**) Modified PP fibers.

Representative SEM images of untreated and modified fibers are shown in Figure 5a and Figure 6. The pristine PP fibers exhibited a relatively smooth and uniform surface. In contrast, the sodium-silicate-modified fibers showed a roughened, irregular morphology, with localized deposits (Figure 6e), which is expected to enhance wettability and chemical affinity towards the cement matrix. This morphological transformation suggests the successful attachment of silicate particles and the formation of a textured surface layer, which is beneficial for improving fiber–matrix adhesion.

Elemental analysis, performed by EDX (Figure 5a,b, Table 5), further confirmed the surface modification. While the untreated PP fibers consisted almost exclusively of carbon, the modified samples exhibited additional signals of silicon and oxygen, consistent with the presence of sodium silicate. For example, Spectrum 1 revealed ~2 wt% Si and ~23 wt% O, while Spectrum 2 contained ~1 wt% Si and ~1.6 wt% O. Minor amounts of potassium and chlorine were also detected, likely originating from residual reagents such as potassium persulfate and ethyl chloroformate.

Although the EDX spectra confirmed the successful deposition of silicate species, the detected silicon contents varied across regions, indicating a non-uniform coating. Such heterogeneous surface coverage is consistent with previous observations in polymer fiber modification studies [28]. Despite this variability, even partial silicate deposition substantially altered the surface chemistry, increasing both the micro-roughness and chemical reactivity of the PP fibers. These characteristics are expected to enhance the interfacial transition zone (ITZ) by facilitating chemical interactions with calcium silicate hydrate (C-S-H) phases during cement hydration. Overall, the SEM/EDX results supported the FTIR evidence, confirming the development of silanol/siloxane functionality on the fiber surface. Although the EDX analysis reveals variations in the silicon percentage across different spectra (as shown in Table 5), this heterogeneity is attributed to the inherent surface roughness of the fibers and the localized nature of the chemical grafting process. The formation of silicon-rich domains at active sites results in micro-scale variations rather than a perfectly uniform monolayer; however, the consistent presence of Si across the samples confirms the successful surface modification.

### 3.2. Cementitious System Properties

#### 3.2.1. Fresh Properties

**Figure 7:**
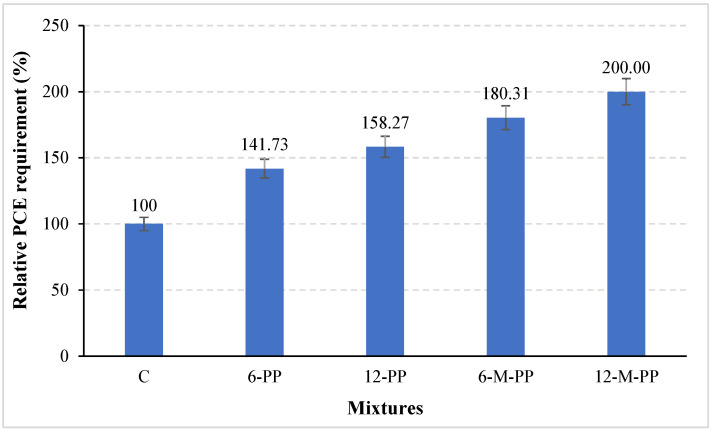
Relative PCE requirement for the target slump-flow value of mixtures.

Figure 7 shows the relative PCE requirements of the mixtures. As observed in the figure, the inclusion of fibers in the mixtures resulted in an increased PCE dosage required to achieve the target slump-flow value, regardless of fiber type or length. Additionally, the PCE requirement for the target slump flow value increased with longer fiber lengths. Increasing the fiber length from 6 mm to 12 mm increased the admixture requirement in the mixtures by 12%. These effects are attributed to fibers enhancing the mixture’s cohesiveness, which negatively impacts workability. Kaya et al. [17] stated that adding fiber to the mixtures reduces the free water in the mixture by adsorbing some of the mixture water to the surface of the fiber, thus weakening the workability.

As seen in Figure 7, the use of modified fiber in the mixtures weakened the workability and increased the admixture requirement of the mixture. While the admixture requirement in mixtures containing unmodified PP fiber increased by up to 58% compared to the control mixture containing no fiber, this rate reached 100% in mixtures containing modified PP fiber.

Modification of PP fibers with sodium silicate increased the roughness of the fiber surface, as seen in the SEM images in Figure 6e. This rough surface may increase the frictional interaction between the fiber particles and reduce the fresh-state mobility. Furthermore, the silicate structures on the surface may undergo secondary reactions with cement ions, potentially contributing to early structuration and further reducing workability.

Instead of negatively affecting fresh-state properties, these products are more likely to contribute to early structuration, which should be interpreted with caution due to the absence of rheological or calorimetric validation.

#### 3.2.2. Compressive and Flexural Strength

**Figure 8:**
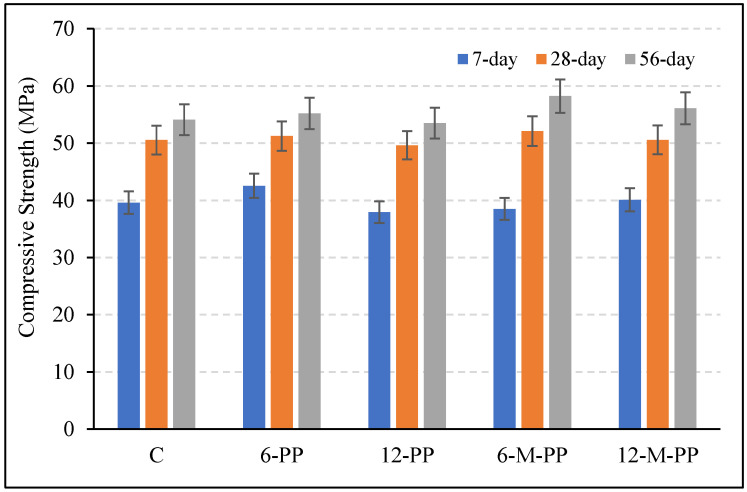
Compressive strength values of the mixtures at 7, 28 and 56 days.

**Figure 9:**
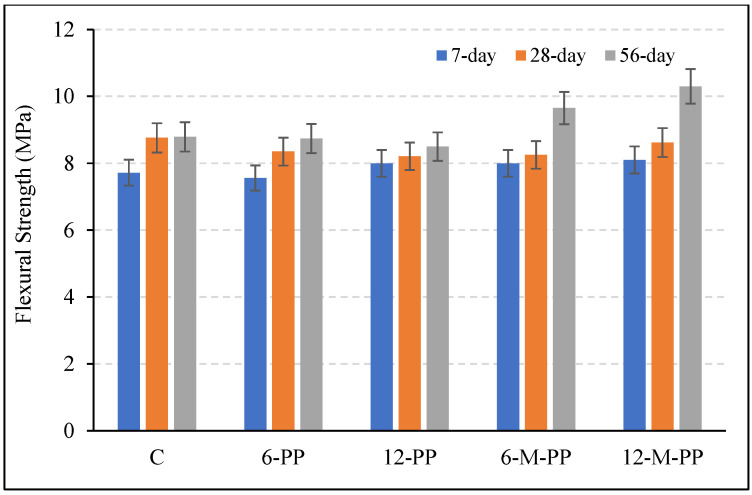
The 7, 28, and 56-day flexural strength values of the mixtures.

Compressive and flexural strength results of the mixtures are shown in Figure 8 and Figure 9. There were increases in compressive and flexural strengths with increasing curing time. When the compressive strengths of the mixtures are examined in Figure 8, it is seen that the 7-, 28-, and 56-day compressive strengths are close to each other. The compressive strength of the sample containing only 6 mm long modified PP fibers was slightly higher than that of the others. Overall, there was no significant change (1–7%) between the compressive strength values of the samples containing PP fiber. The ~1–7% variation indicates a conservative bulk response, which is consistent with an ITZ-dominant mechanism that enhances flexural performance rather than compressive strength.

The 7-, 28-, and 56-day flexural strength values of the mixtures are shown in Figure 9. The flexural strength values of the control and unmodified PP fiber mixtures were similar, with changes limited to approximately 5%. However, while the 7- and 28-day strengths of the modified PP fiber mixtures remained comparable to those of the control, increases of approximately 10% and 17% were recorded at 56 days. This late-age improvement reflects a time-dependent enhancement of the interfacial transition zone (ITZ), where chemically modified fibers promote stronger crack-bridging and bond formation. The flexural gain is therefore attributable not to bulk matrix densification, but to improved fiber-matrix adhesion, which continues to develop with hydration and secondary silicate reactions.

The long-term flexural gains are attributed to the pozzolanic effect of the silicate coating, which likely increases C-S-H formation at the fiber-matrix interface, thereby enhancing chemical adhesion and densifying the ITZ. Future work incorporating permeability tests will be essential to confirm if these interfacial improvements translate into enhanced long-term durability. The rough surface morphology of the modified fibers, as shown in Figure 6e, supports this mechanism by providing an increased surface area for bonding.

Thus, the effect of fibers on the matrix increased. During bending loads, the fibers prevented the development of microcracks under tensile stress and distributed the loads from weaker to stronger regions through their bridging effect [17]. Armandei et al. [29] stated that in fiber cement systems, the fiber, aggregate, and paste phases form regions (colonies) with locally high tensile strength. These regions have higher tensile strengths than traditional aggregate-paste zones. This prevents crack propagation into these zones and diverts the crack trajectory toward the gap between them. In these zones, the fibers and paste phase work together like a strong “armor” around the aggregate, slowing crack propagation.

In mixtures containing modified fibers, the flexural strength of the long-fiber sample was greater than that of the short-fiber sample. The reduced effectiveness of shorter fibers is attributed to their inability to fully participate in the bridging effect, resulting in their detachment from the matrix.

#### 3.2.3. Mechanism

The incorporation of polypropylene fibers—particularly in their modified form—alters both the fresh and hardened properties of the cementitious system through interrelated physical and interfacial mechanisms. In its fresh state, fibers increase the cohesiveness of the mixture and reduce the amount of free water available for flow. This effect, also noted by Kaya et al. [17], arises from the partial adsorption of mixing water onto the fiber surface, leading to decreased workability and, consequently, a higher PCE demand. The increased admixture requirement observed with longer fibers further reflects their greater capacity to restrict flow by creating a more interconnected, resistant network within the paste.

In the hardened state, strength enhancement is governed primarily by improvements in the ITZ. Although early-age strengths of modified fiber mixtures remain similar to those of the control, the pronounced gains at later ages (approximately 10% at 56 days in flexure) indicate a time-dependent improvement in fiber–matrix interaction. The chemically modified surfaces, characterized by their rough and textured morphology, provide increased mechanical interlocking and promote the development of stronger interfacial bonds as hydration progresses. These enhanced interfaces enable more effective crack bridging, delaying microcrack formation and transferring tensile stresses from weaker to stronger regions within the matrix. This behavior aligns with the observations of Kaya et al. [17], who reported that fibers impede microcrack development under bending loads, and with Armandei et al. [29], who demonstrated that fiber–paste–aggregate assemblies form localized high-tensile-strength zones that act as barriers to crack propagation.

Finally, the superior flexural performance of longer modified fibers is attributable to their greater embedment length, which allows them to fully participate in the bridging mechanism. In contrast, shorter fibers are more prone to premature pull-out, limiting their contribution to crack control. Overall, the improved mechanical performance of the modified fiber mixtures stems not from bulk densification of the cementitious matrix, but from the progressive reinforcement of the ITZ and more effective crack-bridging behavior facilitated by the modified fiber surfaces.

## 4. Conclusions

This study investigated the effects of sodium-silicate-based chemical surface modification of polypropylene (PP) fibers on the rheological and mechanical properties of cementitious composites. Based on the experimental findings, several major conclusions can be drawn:

This study demonstrated that sodium-silicate-based chemical modification of polypropylene (PP) fibers effectively enhances their performance in cementitious systems. FTIR, SEM, and EDS results confirmed successful incorporation of silanol and siloxane groups along with increased surface roughness, improving the fibers’ chemical affinity toward cement hydration products. The modified fibers significantly increased superplasticizer demand, doubling the PCE requirement (+100%) relative to the control, while unmodified PP fibers caused a 58% increase.

Although compressive strength was only marginally affected (1–7% variation), the modified fibers produced meaningful improvements in flexural performance, yielding ~10% higher strength at 28 days and ~17% at 56 days. These enhancements are attributed to improved fiber–matrix adhesion and localized ITZ densification induced by the silicate coating.

Overall, the study provides the first evidence that chemically grafting sodium silicate onto PP fibers via a radical-assisted process can strengthen ITZ bonding and flexural behavior in cement-based composites. This modification strategy offers a promising and partially sustainable pathway for producing high-performance fiber-reinforced cementitious materials. Within this context and framework, it becomes practically possible to produce concrete mixtures that utilize lower cement dosages and reduced cement content while still achieving comparable strength levels and mechanical properties to traditional mixtures. Reducing the amount of cement used—which represents the most expensive and costly component of the mixture composition—provides significant advantages in terms of both cost efficiency and economic viability, as well as environmental sustainability and reduced carbon footprint.

Future studies could examine the effects of modifying PP fiber surfaces with different chemicals on the mechanical properties of cementitious systems. Additionally, surface modifications of natural fibers—not only PP fibers—could be explored as a means to enhance both the mechanical performance and durability of cementitious materials.

## Figures and Tables

**Table 1:** Chemical composition, physical and mechanical properties of cement.

Oxide (%)	Cement	Physical Properties		
SiO_2_	18.86	Specific gravity		3.15
Al_2_O_3_	5.71	**Mechanical properties**		
Fe_2_O_3_	3.09	Compressive Strength (MPa)	1-day	14.7
CaO	62.70		2-day	26.80
MgO	1.16		7-day	49.80
SO_3_	2.39		28-day	58.5
Na_2_O + 0.658 K_2_O	0.92	**Fineness**		
Cl^−^	0.01	Specific surface (Blaine, cm^2^/g)		3530
Insoluble residue	0.32	Residue in 0.045 mm sieve (%)		7.6
Loss of ignition	3.20			
Free CaO	1.26			

**Table 2:** Properties of water-reducing admixture.

Tip	Density (g/cm^3^)	Solid Content (%)	pH	Chloride Content (%)	Alkaline Content Na_2_O (%)
**PCE**	1.097	36.35	3.82	<0.1	<10

**Table 3:** Mechanical and physical properties of fibers.

Raw Material	Density (g/cm^3^)	Length (mm)	Tensile Strength (N/mm^2^)	Modulus of Elasticity (N/mm^2^)	Melting Point (°C)
**Polypropylene**	0.91	12	450–700	3000–3500	162

**Table 4:** Material quantities required for the production of 1 dm^3^ mortar mix (g/dm^3^).

**Mix No**	**Cement**	**Water**	**Sand**	**PCE**	**Fiber**	**Slump-Flow** **(mm)**
**C**	550	266.75	1512.50	1.27 (0.23%)	-	182
**6-PP**	550	266.75	1499.05	1.80 (0.33%)	4.550	200
**12-PP**	550	266.75	1499.05	2.01 (0.37%)	4.550	184
**6-M-PP**	550	266.75	1499.05	2.29 (0.42%)	4.550	185
**12-M-PP**	550	266.75	1499.05	2.54 (0.46%)	4.550	195

**Table 5:** Normalized mass concentration (%) table of PP fiber treated with sodium silicate.

Spectrum	Carbon	Nitrogen	Oxygen	Sulfur	Silicon	Chlorine	Potassium
Spectrum 1	48.73	18.67	23.04	0.39	2.00	7.05	0.12
Spectrum 2	91.34	4.67	1.64	0.20	1.04	0.17	0.02

## Data Availability

The original contributions presented in this study are included in the article. Further inquiries can be directed to the corresponding author.

## Abbreviations

PP	Polypropylene
ITZ	Interfacial Transition Zone
C-S-H	Calcium Silicate Hydrate
Ca(OH)_2_	Calcium Hydroxide
Na_2_SiO_3_	Sodium Silicate
KPS	Potassium Persulfate
K_2_S_2_O_8_	Potassium Persulfate
C_2_H_5_OCOCl	Ethyl Chloroformate
CIPP	Chlorination of Polypropylene
CIPP-SiO_2_	Silica Functionalization of Polypropylene
PCE	Polycarboxylate Ether
FT-IR	Fourier Transform Infrared Spectroscopy
SEM	Scanning Electron Microscopy
EDX	Energy Dispersive X-Ray Spectroscopy
EDS	Energy Dispersive Spectroscopy
PPE	Personal Protective Equipment
bwob	By Weight of Binder
rpm	Revolutions Per Minute
wt%	Weight Percent
CAS	Chemical Abstracts Service
